# Optical Metrology under Extreme Conditions

**DOI:** 10.1155/2014/263603

**Published:** 2014-05-26

**Authors:** Xide Li, Giancarlo Pedrini, Yu Fu

**Affiliations:** ^1^Department of Engineering Mechanics, Tsinghua University, Beijing 100084, China; ^2^Institut für Technische Optik, University of Stuttgart, Pfaffenwaldring 9, 70569 Stuttgart, Germany; ^3^Temasek Laboratories, Nanyang Technological University, Research Techno Plaza, 50 Nanyang Drive, Singapore 637553

Optical metrology provides full field, noncontact, precise measurement of various physical parameters of materials, structures, and devices. These properties include kinematic parameters (displacement, velocity, and acceleration), deformation parameters (strains, curvature, and twist), surface parameters (shape and roughness), and mechanical properties of materials (Young's modulus, Poisson's ratio, etc.). Researchers have developed many delicate optical measurement techniques and methods, such as photoelasticity, holographic interferometry, speckle metrology, Moiré methods, fiber sensing, laser Doppler vibrometry and velocimetry, and computer-vision-based techniques. However, new requirements arise with the recent development in fundamental research and industry applications to fulfill nondestructive and precise measurement under extreme conditions, such as high temperature or pressure, micro/nanoscale samples, large-scale curved structures, and transient events.

It is our pleasure to have this opportunity to present the readers of The Scientific World Journal (SWJ) to some of the current research being performed in optical techniques and image processing under some extreme conditions. There are 10 papers in this special issue, which cover a broad range of topics from microscopic image analysis to nanoscale strain field research, from interfacial mechanical analysis to Raman spectroscopy detection, and from high temperature measurement to large-scale structure optical tracking and dynamical or transient optical metrology. All of the published papers are peer-reviewed.

*Xide Li* *Xide Li* *Giancarlo Pedrini* *Giancarlo Pedrini* *Yu Fu* *Yu Fu*

